# Carcinoid tumour of the appendix in children: a case report

**DOI:** 10.1186/1757-1626-1-136

**Published:** 2008-09-01

**Authors:** Efstratios Christianakis, Nikolaos Paschalidis, Maria Chorti, Georgios Filippou, Spiros Rizos, Dimitrios Filippou

**Affiliations:** 1 Department of Paediatric Surgery, Penteli's Children Hospital, Palaia Penteli, Athens, Greece; 2 Department of Pathology, Athens General Hospital "Sismanoglio", Melissia-Athens, Greece; 3 First Department of General Surgery, Piraeus General Hospital "Tzaneio", Piraeus-Athens, Greece; 4 Department of Anatomy, Histology and Embryology, Nursing Faculty, University of Athens, Athens, Greece

## Abstract

Carcinoids are the most common tumours of the appendix. These tumours show prevalence in white children. The clinical presentation of the appendiceal carcinoids is similar to that of acute appendicitis, although in many cases the tumour is diagnosed incidentally during an operation. The diagnosis should be confirmed histologically. The prognosis in patients with local disease is excellent. In small lesions isolated appendicectomy is considered as the most appropriate treatment, while in larger lesions right colectomy should be performed. We report a case of a carcinoid tumour in the tip of the appendix of a thirteen year old girl which was diagnosed intraoperatively. The patient received isolated appendicectomy due to the small size of the lesion. Ten years after the operation there is no evidence of recurrence or metastases, and the patient is considered free of disease.

## Background

Carcinoid tumours (CTs) are the most common neoplasm of the appendix [1,2]. The overall incidence of carcinoid tumours has been estimated to 1 to 2 cases per 1000 appendectomies in surgical specimens [3]. CTs are discovered usually during the course of another procedure. In children the tumour is usually smaller than 2 cm in diameter [4]. A carcinoid tumour of the appendix may cause pain in lower abdominal quadrant, similar to the pain of acute appendicitis. The diagnosis should be confirmed histologically.

In our case a carcinoid tumour in the tip of the appendix presented as acute appendicitis. It was the only appendiceal tumour throughout a total of 1540 appendicectomies, performed during the last eighteen years.

## Case presentation

**Figure 1:**
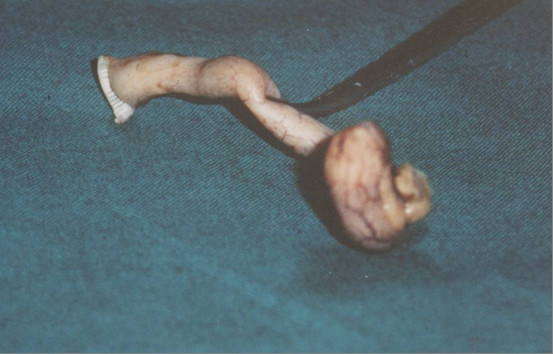
Intraoperatively we defined a solid, moderately hard, elastic and yellowish mass on the appendix tip, with diameter 1,0 cm.

**Figure 2:**
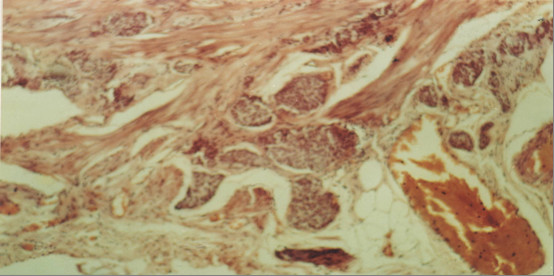
The fatty tissue of the appendiceal mesenterium is invaded by cancer cells.

A 13-years-old female patient presented complaining for abdominal pain in right lower quadrant (RLQ), nausea and decreased appetite the last two days. Physical examination revealed a healthy looking female with a mildly elevated temperature 38°C, blood pressure 110/80 mmHg and pulse rate 95/min. The patient showed no sings of acute abdomen suggestive of acute appendicitis, as direct and rebound tenderness in the RLQ. Rectal examination revealed a mild tenderness in the RLQ, but no blood or palpable masses were observed. Hematocrite was 40%, white blood cells 13700/mm with neutrophil prevalence 87%, platelets 175000/mm, erythrocytes sedimentation rate 55 mm/h, while the rest blood analysis was normal. Abdominal ultrasound revealed appendiceal inflammation with transverse appendix diameter to be 9 cm. The patient was operated for acute appendicitis. The appendix found inflamed with distension of the tip, which was palpated as a solid, moderately hard, elastic and yellowish mass with diameter 1 cm.(Figure 1) No local or regional enlarged lymph nodes were found. The histological examination revealed a typical CT of the appendix and tumour-free margin in all specimens. We used haematoxylin-eosin as staining method. The tumour was consisted of small homogenic neoplasmatic cells that were arranged in islet formation and infiltrated few areas of the muscular layer. The mitotic activity was insignificant. There was a coexisting acute appendicitis and periappendicitis. (Figure 2)

5-hydroxyindoleacetic acid (5-HIAA) which was measured postoperatively was normal at 3,2 mg/24 h. The patient's postoperative course was uncomplicated and she was discharged the 4^th ^day. Ten years later the patient is free of local or metastatic disease.

## Discussion

Carcinoid tumours of the appendix are relatively uncommon neoplasms. Although are considered rare pathology in children, these are the most frequent tumours of the gastrointestinal tract in childhood and adolescence [5]. They are usually benign neoplasm and the uncommon occurrence of metastasis is related to the primary tumour size and depth [6].

The reported incidence of appendiceal carcinoids in several studies ranges from 0.08 to 0.7% in surgical specimens [5-8]. Willox in 1964 suggested that 0.2–0.5% of surgically removed appendices in children contained CT [9]. Doede et al [8] and D' Aloe et al [10] estimated that CTs of the appendix occur in 1:100,000 to 169:100,000 children, respectively. These data illustrate the rarity of CT and explain the low rate of suspicion of this disease [11]. The tumour is more common in white females with a mean age of 12 to 13 years [12,13]. Some authors have been reported CT in children 3 years of age.

The clinical presentation of the appendiceal carcinoids is similar to that of acute appendicitis, but in some cases the disease is incidentally found during surgery performed for another diagnosis or problem. Recurrent episodes of abdominal pain were reported in many cases may indicate partial obstruction of the appendiceal lumen by a tumour [12]. In two reported cases the patients presented with clinical signs of peritonitis without previous episodes of acute abdominal pain [12]. Symptoms of the carcinoid syndrome as flushing, diarrhoea, cardiac disease have been rarely reported and usually associated with liver or retroperitoneal metastases [8,14]. In these cases an increased urine excretion of 5-HIAA has been documented and in monitoring disease progression [8,14]. Our patient had no symptoms related to carcinoid syndrome, neither metastatic spreads nor 5-HIAA increased excretion. The majority of carcinoid tumours are discovered during the histological examination of the surgical specimen incidentally and rarely suspected before this examination [15].

At present, the site and the size rather than the depth, are used for the assessment of the tumour [4]. In the 75% of cases the tumour is localized at the apex of the appendix, in 20% and 5% affect the mid portion and the base respectively [13]. The tumour's median diameter is 6 mm [4,13]. In our case the diameter of the tumour was 1 cm. Generally, carcinoid tumours located at the tip of the appendix and measuring less than 10 mm usually are mimicking the clinical presentation of acute appendicitis, while tumours measuring more than 20 mm and located at the base of the appendix may present with clinical signs of peritonitis [12,16]. The prognosis is directly related to the tumour's size. If the tumour is smaller than 2 cm and has perforated the serosa, the treatment of choice is appendectomy, whatever the location. Other reports suggest that neoplasms with these characteristics do not tend to relapse. Tumours measuring 2 cm or more in diameter may have widespread metastases upon detection. The invasive properties of these tumours are well-known, but the presence of lymph node metastases is reported in only 4% to 5% of paediatric cases. The prognosis is excellent [12]. It should be mentioned that carcinoid is not infrequently associated with MEN1 and loss of 11q, sometimes independently of the MEN1 gene (11q13), suggesting loss of MEN1 or another tumour suppressor gene is responsible for the condition. However, this is usually isolated to foregut carcinoids [17].

Metastasis of an appendiceal carcinoid is very rare in children probably because most reported tumours in this age group are small and less aggressive. In this case the tumour extended thought the entire diameter of the appendix and involved all layers of the wall to the overlying serosa surface without distant metastasis.

The treatment of the carcinoid tumours in the appendix depends to the size and the site of the tumour. Tumours smaller than 2 cm can be adequately treated by appendectomy, while right hemicolectomy is recommended for pediatric patients with appendiceal carcinoid tumours larger than 2 cm, especially when the mesoapendix is involved or in cases with residual tumours at the margin of resection [10]. Our patient had an appendiceal carcinoid tumour of 1 cm in size, respectively with tumour free-margin in all specimens; simple appendectomy was considered the adequate treatment for this patient.

## Conclusion

In conclusion, CTs are the most common tumours of the appendix. In children, they occur more commonly in white females with a mean age of 13 years. The clinical presentation of the CT is similar to acute appendicitis, but the CT can be an incidental finding during surgical procedures other than appendicectomy. CT was diagnosed on histological examination of the removed appendix. The site and the size of the tumours rather than the depth, are used for the assessment of the CT. Localized disease has an excellent prognosis. Patients with metastatic CT fare poorly. Single appendicectomy is considered the appropriate treatment, while right colectomy is indicated in tumour larger than 2 cm. We report a case of an appendiceal CT in a 13 year old girl with diameter 1 cm which was treated with isolated appendectomy. The patient is disease free of ten years follow-up. Clinical awareness and early diagnosis of CT of the appendix may significantly decrease morbidity and mortality.

## Abbreviations

CTs: Carcinoid tumours; CT: Carcinoid tumour; cm: centimeters; RLQ: right lower quadrant; mm: millimeter; mm/h: millimeters per hour; °C: Celcious degrees; min: minutes; 5-HIAA: 5-hydroxyindoleacetic acid; mg: milligrams.

## Competing interests

The authors declare that they have no competing interests.

## Authors' contributions

EC participated in the patients treatment, had the concept of the case report and contributed in the first draft, NP participated in the first draft and in the revisions, MC participated in the diagnosis of the case and in the presentation of its pathology, GF contributed in writing the paper, SR contributed in writing the paper, and DF participated in patient;s treatment, co-wrote the first draft and performed all the revisions.

## Consent

Written informed consent was obtained from the patient for publication of this case report and accompanying images. A copy of the written consent is available for review by the Editor-in-Chief of this journal.
